# Health Tract, No. 73

**Published:** 1862-06

**Authors:** 


HEALTH TRACT, No. 73

### BREAD AND BUTTER "TEA."

As usually ground, one hundred pounds of wheat yield from seventy-eight to eighty pounds of flour, but when "hulled" before grinding, it yields from ninety to ninety-five pounds. Connoisseurs of the table know that when the bare outside, thin skin of the potato is removed, the most nutritious part of the vegetable is the one fourth of an inch of the outer portion of it, more nutritious than the whole remaining inner part or core; so in wheat, the most nutritious part of the grain is that which is immediately outside of the kernel and adheres to the hull; hence the ten per cent of flour lost in the common mode of grinding, is the most nutritious portion of it. It is in this part, also, that weevils and other animalculæ lay their eggs, and such flour does not keep, while that ground of hulled wheat will, under the same circumstances, keep for years, looking fully as well, and is quite as agreeable to the taste.

Butter.—The best temperature for getting butter out of milk quickly, is sixty-eight degrees, ten times quicker than if at fifty-four. If cream is churned, it should be about sixty-eight degrees.

Tea.—Take pure soft water, make it boil rapidly, and the moment it has boiled, make the drawing of tea in the usual way, thus retaining the gas in the water, which gives that lifelike and sparkling quality so contradistinguished from "flat," "dead" drinks—dish-water, for example.

It is very certain that several years would be added to civilized life if, from infancy to age, nothing were eaten after dinner but a bowl of pure, fresh, sweet milk, and cold, light, or hot corn-bread for children; and for those over a dozen years of age, the "supper" should be made of stale, coarse, "light bread," with fresh butter made of rich milk, and a cup of hot tea; for children, made of boiled milk and hot water, half-and-half, with sugar, adding green or black tea in later years. The effect of such a "supper" would be to allow the stomach to rest at night, with the other parts of the body, by which it would be laying up a stock of strength to be expended on a hearty breakfast. The most unobservant know that a very hearty supper will inevitably be followed by a disturbed, dreamy, or otherwise unrefreshing sleep. Just as certain as any thing in nature can be certain, will a bad night's rest be succeeded by a weary waking up, with an entire day following of more or less discomfort; an irritability of mind or depression of spirits, while the body lacks that animation, vivacity and elasticity so inseparable from vigorous health, and without which life is pleasureless, if not actually burdensome. What an inconsideration it is, that for the small and literally momentary pleasure of swallowing a little "relish" of chipped beef, pound-cake, preserves, hot rolls, or ginger-bread, all of which tempt and lead on the appetite to excesses, we incur the discomforts just alluded to, at the expense of a sound sleep, a glad awakening, a glorious appetite, and a pleasurable day.

